# Exosomal PD-L1 detection in cancer predictive biomarker for response to immune checkpoint blockade therapy

**DOI:** 10.3389/fimmu.2025.1603855

**Published:** 2025-07-03

**Authors:** Tetsuichi Kansha, Xiaojuan Ma, Hao Wang, Xiaotong Yu, Ying Song, Zhengyang Guo, Jiagui Song, Lixiang Xue, Jianling Yang

**Affiliations:** ^1^ Center of Basic Medical Research, Institute of Medical Innovation and Research, Peking University Third Hospital, Beijing, China; ^2^ Cancer Center of Peking University Third Hospital, Peking University Third Hospital, Beijing, China; ^3^ Beijing Key Laboratory for Interdisciplinary Research in Gastrointestinal Oncology (BLGO), Peking University Third Hospital, Beijing, China; ^4^ Biobank, Institute of Medical Innovation and Research, Peking University Third Hospital, Beijing, China

**Keywords:** exosomal PD-L1, cancer biomarkers, early detection, liquid biopsy, immune checkpoint blockade

## Abstract

Programmed death-ligand 1 (PD-L1) carried by tumor-derived exosomes has emerged as a critical mediator of immune evasion and resistance to immune checkpoint blockade therapy. Unlike membrane-bound PD-L1, exosomal PD-L1 is systemically distributed and capable of suppressing T cell activity at distant sites. This review summarizes the current understanding of exosomal PD-L1 biogenesis, its immunosuppressive mechanisms, and its clinical relevance across multiple cancer types. We highlight its potential as a non-invasive biomarker for predicting therapeutic response and monitoring disease progression. Compared with tissue-based PD-L1 assessment, exosomal PD-L1 offers advantages in accessibility and dynamic reflection of tumor immune status. However, challenges remain regarding standardization of detection methods and clinical interpretation. Future directions include the integration of exosomal PD-L1 profiling into immunotherapy decision-making and the development of therapeutic strategies targeting exosome secretion. These insights may contribute to overcoming resistance in immunologically inert tumors and advancing precision oncology.

## 1 Introduction

Immune checkpoint blockade (ICB) therapies, particularly those targeting the programmed cell death protein 1 (PD-1) and its ligand PD-L1, have revolutionized cancer treatment by restoring anti-tumor immunity in a subset of patients (1). Despite their success, less than 30% of patients can achieve durable clinical benefit, and both primary and acquired resistance remain major obstacles to broader efficacy (2–5). This underscores a critical need for reliable, minimally invasive biomarkers that can predict therapeutic response and guide personalized immunotherapy strategies.

To date, biomarkers such as PD-L1 expression in tumor tissues, tumor mutational burden (TMB), and microsatellite instability (MSI) have been investigated with varying predictive value. However, the clinical application of these markers is limited by tumor heterogeneity, dynamic expression, sampling bias, and the invasive nature of tissue biopsies (6–8). Furthermore, pan-cancer analyses have demonstrated wide variability in response rates to anti-PD-1/PD-L1 therapies—even within the same tumor type—suggesting that existing tissue-based indicators do not fully capture systemic immune escape mechanisms (9).

Exosomal PD-L1 (exo-PD-L1), a membrane-bound form of PD-L1 secreted via tumor-derived exosomes, has emerged as a promising biomarker with distinct advantages (9). Exosomes are small extracellular vesicles that facilitate intercellular communication by transporting bioactive molecules, including PD-L1, to immune cells (10, 11). Unlike static tumor PD-L1 measurements, exo-PD-L1 levels in peripheral blood provide a dynamic and systemic snapshot of tumor-mediated immune suppression (12). Elevated circulating exo-PD-L1 has been associated with poor prognosis, resistance to ICB, and increased tumor burden across various cancers (9, 13–15).

In this review, we explore the biological mechanisms underlying exo-PD-L1 secretion and its immunosuppressive role in the tumor microenvironment. We summarize current detection strategies and assess the clinical significance of exo-PD-L1 as a predictive biomarker across cancer types. Finally, we highlight future research directions and potential applications of exo-PD-L1 in enhancing ICB precision, overcoming resistance, and advancing personalized cancer immunotherapy.

## 2 Biogenesis and immune regulation of exosomal PD-L1

### 2.1 Biogenesis and secretion of exo-PD-L1

Exosomes are biologically active, lipid bilayer nano-vesicles. Current evidence suggests that exo-PD-L1 originates from the plasma membrane. Consequently, it is widely believed that early endosomes formed via cellular membrane endocytosis serve as the source of exo-PD-L1.

The process begins with the inward invagination of the parent cell’s plasma membrane, leading to the closure and formation of early endosomes. Within these early endosomes, intraluminal vesicles (ILVs) are generated through multiple inward budding events, ultimately resulting in the formation of mature multivesicular bodies (MVBs) containing ILVs. During MVB formation, certain endosomal proteins and other cellular components, not destined for lysosomal degradation, are selectively sorted into ILVs. Upon maturation, MVBs fuse with the cellular membrane, releasing the vesicles as exosomes, which typically range in size from 30 to 150 nm (10, 11). They are secreted by all active cells and are found in various bodily fluids (16). Exosomes’ cargo typically includes a diverse array of RNA, DNA, proteins, miRNA, metabolites, and other bioactive molecules (17).These cargoes mediate intercellular signaling, information exchange, and immune modulation by transporting their contents to recipient cells (10, 18–22).

Importantly, during the formation of intraluminal vesicles (ILVs), membrane proteins such as PD-L1 are incorporated with preserved topological orientation. This means that transmembrane proteins located on the plasma membrane of the parent cell are embedded into the ILV membrane such that their extracellular domains face the lumen of the ILV. Upon exosome release, the ILV becomes an exosome, and its membrane orientation flips relative to the cytoplasm, thereby exposing PD-L1 on the outer surface of the exosome. This membrane topology conservation ensures that exo-PD-L1 retains its ability to engage PD-1 receptors on recipient T cells, thereby mediating immunosuppressive interactions post-secretion (9, 23, 24).

### 2.2 Function of exo-PD-L1

Given the conserved membrane orientation of PD-L1 on exosomal surfaces, exo-PD-L1 is functionally positioned to interact with PD-1 on T cells and other immune populations. Interaction of exo-PD-L1 with PD-1 on immune cells triggers PD-1-mediated intracellular signaling, inhibiting PI3K-AKT and MAPK pathways, thereby restricting T cell proliferation, activation, and survival (6–8). Prolonged exposure to elevated levels of exo-PD-L1 leads to T cell exhaustion, impacting long-term immune responses and reducing responsiveness to tumors, thereby facilitating immune escape. The interaction between TCR and MHC molecules is essential for the first signal required to induce T cell activation. On the exosome surface, the presence of MHC-I can enhance PD-L1-induced T cell dysfunction. Therefore, exo-PD-L1 can more effectively induce T cell dysfunction (8, 14, 25). Removing exosomal PD-L1 can effectively enhance the sensitivity of mouse tumor models to anti-PD-L1 immune checkpoint therapy (4, 9, 13). This finding suggests potential therapeutic strategies, which will be discussed in the next section regarding the mechanisms of immune suppression induced by exosomal PD-L1.

**Figure 1:**
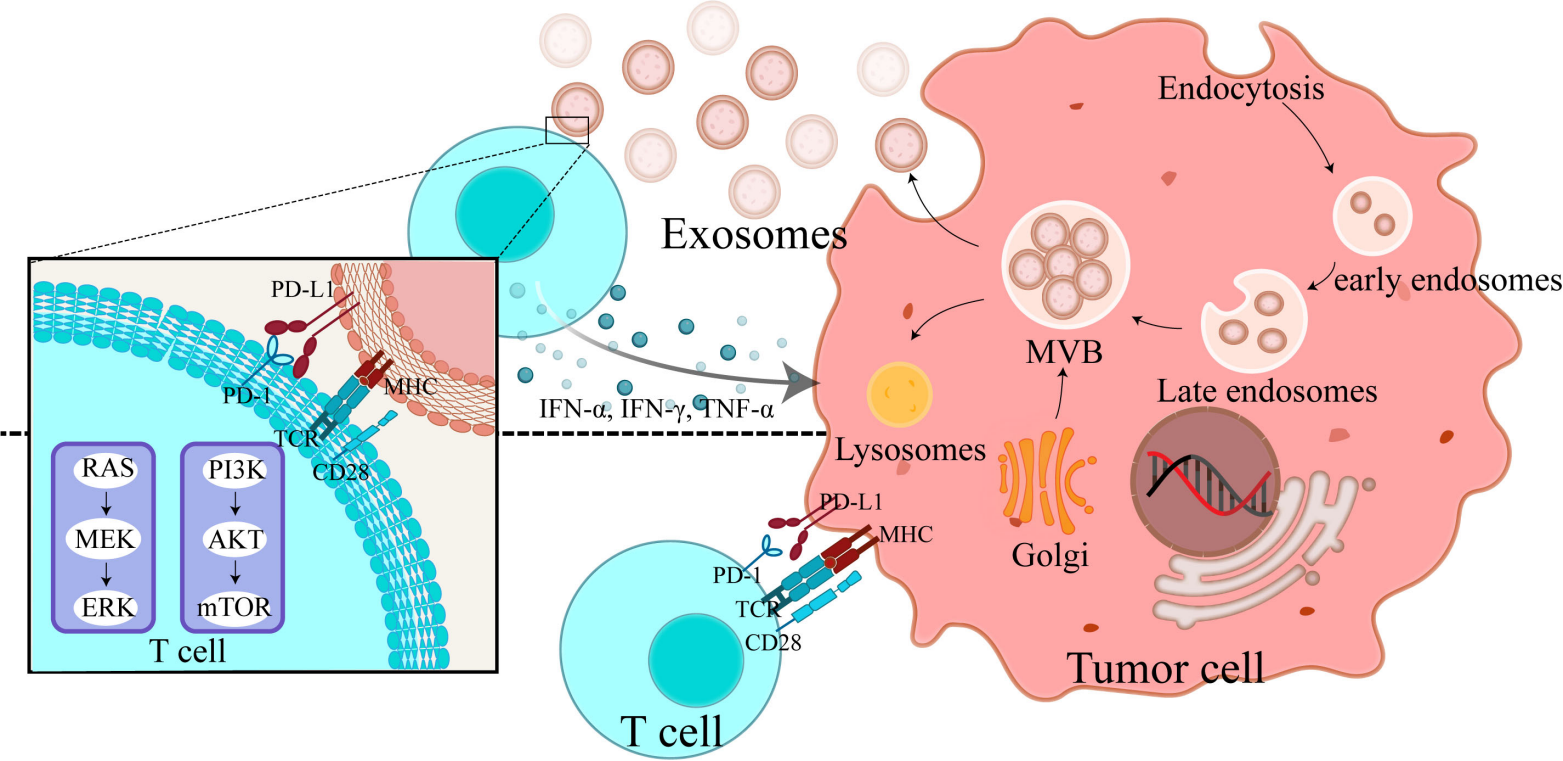
Exosomal PD-L1 release mechanism and its impact on T cell function: tumor cell-mediated immune evasion. This figure provides a detailed depiction of the exo-PD-L1 release mechanism from tumor cells and its impact on T cell function. It depicts exosome biogenesis, starting from plasma membrane invagination, progressing to the formation of multivesicular bodies, and culminating in the release of PD-L1-containing exosomes. Additionally, it shows how cytokines—IFN-α, IFN-γ, and TNF-α—stimulate exosome release and enhance PD-L1 expression on tumor cells. Two key interactions are highlighted (1): the direct binding of PD-L1 on tumor cells to T cells, resulting in T cell inhibition, and (2) the interaction of T cells with PD-L1 on exosomes, leading to immune suppression over longer distances. This figure demonstrates how exo-PD-L1 contributes to immune evasion and how cytokine stimulation affects its release and expression.

Furthermore, extensive experiments demonstrate that interferon-gamma significantly increases exo-PD-L1 release. Exo-PD-L1 release is considered as an immune evasion mechanism in response to interferon-gamma secreted by CD8+ T cells, macrophages, and natural killer cells. *In vitro* studies show that exo-PD-L1 also reduces secretion of interferon-gamma, tumor necrosis factor-alpha, granzyme B, and perforin from T cells (4, 13–15). This suggests that tumor cells can counteract CD8+ T cell function during their effector phase by exo-PD-L1, inhibiting cytokine production and cytotoxic granule exocytosis without requiring direct cell-to-cell interactions (**Figure 1**). Recent studies have revealed that tumor-derived exosomes carrying PD-L1 can promote T cell senescence through lipid metabolism reprogramming (26). This process involves metabolic shifts in T cells that accelerate their aging process, impairing their ability to produce cytokines and perform cytotoxic functions. These changes contribute to long-term immune suppression, further enhancing tumor immune evasion (26–28).The lipid metabolism alterations induced by exo-PD-L1 result in T cell dysfunction and senescence, diminishing their effectiveness in combating tumors. By promoting T cell aging, exo-PD-L1 not only suppresses immediate immune responses but also impairs the ability of T cells to mount sustained anti-tumor immunity (26, 29). This new mechanism of T cell senescence adds to the understanding of how tumors exploit exosomal PD-L1 to evade immune surveillance, underscoring the potential of targeting this pathway for therapeutic intervention.

In summary, exo-PD-L1 mediates multifaceted immunosuppressive effects, including the inhibition of T cell activation, the promotion of exhaustion and senescence, and the suppression of cytokine secretion and cytotoxic granule release. Emerging evidence also suggests that exo-PD-L1 may influence the function of antigen-presenting cells and regulatory T cells, warranting further investigation.

### 2.3 Factor regulating exo-PD-L1

Tumor heterogeneity is a major factor influencing the effectiveness of exo-PD-L1 as a reliable biomarker for predicting immunotherapy outcomes. This heterogeneity can manifest at various levels, including tumor type, immune cell infiltration, genetic mutations, and even the tumor microenvironment (TME), all of which can affect exo-PD-L1 expression. The levels of exo-PD-L1 in circulation may vary significantly due to these factors, limiting its predictive value across different cancer types and patients (30).

For example, ovarian cancer, often referred to as a ‘cold tumor’, is characterized by low immune cell infiltration, particularly T cells. As a result, exo-PD-L1 levels in ovarian cancer patients may be lower than in cancers with higher immune infiltration, such as melanoma or non-small cell lung cancer (NSCLC). Similarly, in tumors with significant genetic mutations or TME dysregulation, the regulation of PD-L1 expression on both tumor cells and exosomes may vary, leading to inconsistent levels of exo-PD-L1 in the blood.

Additionally, the tumor’s immune microenvironment plays a crucial role in modulating exo-PD-L1 secretion (31). In cancers with an immunosuppressive TME, such as pancreatic cancer, high levels of cytokines and other immune modulators might promote the secretion of exo-PD-L1 by tumor cells and immune cells. Conversely, tumors with a more pro-inflammatory TME might show a different pattern of exo-PD-L1 expression.

Moreover, patient-specific factors, including genetic predispositions, prior treatments, and the overall immune status of the individual, can contribute to differences in exo-PD-L1 levels (9). For instance, patients with a history of autoimmune diseases or those who are immunosuppressed might exhibit altered exo-PD-L1 dynamics, further complicating the biomarker’s predictive value.

### 2.4 Exo-PD-L1 in tumor progression and immunotherapy monitoring

The cargo of extracellular vesicles (EVs) provides a holistic snapshot of the patient’s immune status (8, 13, 32). exo-PD-L1 correlates with tumor progression in various cancers such as melanoma, breast cancer, head and neck squamous cell carcinoma, and glioblastoma (9, 13, 24, 33). Notably, elevated exo-PD-L1 expression is significantly associated with advanced tumor stages, highlighting its pivotal role in fueling tumor growth and metastasis. Previous studies utilizing various murine models have consistently shown that the introduction of tumor-derived EVs amplifies the metastatic propensity of primary tumors and exacerbates overall tumor burden (34–36).

Therefore, targeting exogenous exosomes may provide a novel strategy to overcome tumor resistance to anti-PD-L1 therapies (37). Monitoring circulating exo-PD-L1 levels serves as a biomarker for tumor response to immunotherapy. The dynamic changes in exo-PD-L1 levels during treatment could be used to predict the likelihood of a sustained immune response and inform adjustments to therapy, ultimately optimizing individualized treatment regimens (38).

## 3 Exo-PD-L1 in the tumor immune suppression

The tumor microenvironment (TME) is a complex ecosystem composed of various immune cells, stromal components, and signaling molecules that collectively influence tumor progression and immune escape (31, 39). Exosomal PD-L1 (exo-PD-L1) has become a key player in mediating immune suppression within the TME (9, 40). Secreted by both tumor cells and infiltrating immune cells, exo-PD-L1 can modulate the immune response by transferring immune checkpoint molecules to surrounding immune cells, primarily T cells. This process contributes to immune evasion and aids tumor progression by inhibiting T cell activation and function (9, 14).

### 3.1 Exo-PD-L1 and immune suppression

Exo-PD-L1, primarily released by tumor cells and tumor-associated macrophages (TAMs), plays a pivotal role in shaping the immune landscape of the TME. Tumor-derived exosomes carry PD-L1 to the surface of immune cells, such as T cells and dendritic cells, and bind to the PD-1 receptor, effectively inhibiting their activity. By doing so, exo-PD-L1 fosters a suppressive immune microenvironment that prevents T cells from recognizing and attacking tumor cells. This immune evasion mechanism is crucial for tumor survival, particularly in the context of immunotherapy resistance (39).

In addition to its effects on T cells, exo-PD-L1 can also influence the activity of other immune modulators within the TME. For example, tumor-derived exosomal PD-L1 has been shown to promote the polarization of macrophages toward the M2 (immunosuppressive) phenotype, thereby contributing to a more suppressive tumor immune microenvironment and facilitating immune escape (41, 42). This reprogramming is linked to the secretion of cytokines such as TGF-β, which suppresses T cell activity and promotes tumor growth (43, 44). TAM-derived exosomes, containing miR-21 and other microRNAs, can further exacerbate immune suppression by inhibiting T cell proliferation and inducing the recruitment of regulatory T cells (Tregs) to the tumor site (45, 46).

### 3.2 Exo-PD-L1 and resistance to immunotherapy

The ability of tumors to evade immune surveillance by secreting exo-PD-L1 is a significant factor in the development of resistance to immune checkpoint inhibitors (ICIs), such as anti-PD-1/PD-L1 therapies. While ICIs are designed to block the PD-1/PD-L1 interaction and restore T cell function, the presence of exo-PD-L1 in the TME can counteract these therapies by maintaining an inhibitory signaling axis that diminishes T cell responses. Exo-PD-L1 thus represents a critical mechanism of “immune checkpoint resistance” in the context of ICI treatment. Additionally, exo-PD-L1 can act as a decoy by competitively binding anti-PD-L1 antibodies, thereby reducing their availability to block membrane-bound PD-L1 on tumor cells. This mechanism contributes to the limited efficacy of checkpoint inhibitors in some patients (14, 47).

Moreover, exosomes from tumor cells can also promote immune suppression by facilitating the transfer of other immune checkpoint molecules such as VISTA (V-domain Ig-containing suppressor of T cell activation), which can act synergistically with PD-L1 to inhibit T cell function (48, 49). This underscores the complexity of the immune evasion mechanisms in the TME, where multiple immune checkpoints are concurrently regulated, often rendering ICI therapy less effective.

### 3.3 Advantages of exo PD-L1 as a predictive biomarker for ICB response

While section 2.5 presented clinical evidence supporting exo-PD-L1 as a biomarker for tumor progression and immunotherapy response, this section highlights the comparative advantages of exo-PD-L1 over other circulating and tissue-based biomarkers, such as IHC-PD-L1, ctDNA, and soluble PD-L1.

The limited efficacy of current biomarkers in predicting immune checkpoint blockade (ICB) response reflects the complexity of tumor-immune interactions, encompassing genomic, spatial, and immunologic heterogeneity. Conventional markers—such as PD-L1 immunohistochemistry (IHC), tumor mutational burden (TMB), and microsatellite instability (MSI)—offer static, region-specific snapshots that often fail to represent dynamic immune status or therapeutic adaptation (33–36)

Liquid biopsy approaches have sought to overcome these limitations. While circulating tumor DNA (ctDNA) provides mutational insights, it does not reflect real-time immune suppression and is affected by tumor shedding kinetics (37). Soluble PD-L1 (sPD-L1), though easily detectable in plasma, lacks stability and functional specificity due to its unbound, cleaved nature (38, 39).

By contrast, exosomal PD-L1 (exo-PD-L1) represents a membrane-bound, functionally active form of PD-L1, selectively packaged via Rab27a and ESCRT-dependent pathways (9). Exosomes preserve PD-L1 integrity and extend its immunosuppressive reach beyond the tumor, notably trafficking to lymph nodes and modulating systemic immunity.

Clinically, high baseline exo-PD-L1 levels correlate with poor prognosis and early resistance to ICB in NSCLC and melanoma, while dynamic increases post-treatment predict durable responses—even before imaging confirmation (9). Unlike static biomarkers, exo-PD-L1 integrates upstream oncogenic signals (e.g., IFN-γ/JAK-STAT), microenvironmental factors (e.g., hypoxia), and downstream immune suppression, serving as both a functional and temporal indicator of tumor immune escape (40).

In sum, exo-PD-L1 is a mechanistically distinct biomarker with potential for real-time monitoring and stratification in precision immunotherapy (40).

## 4 Clinical implications of exosomal PD-L1 across cancer types

In recent years, immune checkpoint blockade (ICB) therapies targeting CTLA-4, PD-1, and PD-L1 have shown promising efficacy in various cancers, including melanoma, NSCLC, and renal cell carcinoma (50–53). However, sustained responses are observed in fewer than 30% of patients, with minimal benefit in malignancies like ovarian and prostate cancer (54, 55) (**Table 1**).

**Table 1:** The relationship between exo-PD-L1 and prognosis in different tumors.

Cancer type	n	Conclusion	Method	Reference
Melanoma	44	Metastatic melanoma’s exoPD-L1 > healthy donors, post-treat rise exo-PD-L1 levels show anti-PD-1’s response.	ELISA	(9)
Osteosarcoma	67	Higher initial exoPD-L1 levels, poorer disease-free survival and worse overall survival.	Immunogold labeling and ELISA	(56)
NSCLC,GC, HNSCC, CC, RCC,HCC,CHC,EC,DC and Melanoma	23,2,1,3,2,1,1,2,5,1,1	Pre/Post-treat ExoPD-L1 varies in responders & non-responder.	ELISA	(32)
Melanoma	100	Treat-change, but not baseline ExoPD-L1, affect tumor response.	ELISA and IHC	(38)
NSCLC	24	Number of PD-L1+ exosomes ties to tumor tissue’s PD-L1.	FCM	(14)
NSCLC	85	exo-PD-L1 levels are positively correlated with tumor size and higher metastasis.	ELISA and IHC	(58)
NSCLC	51	exo-PD-L1 fold change predicts NSCLC treatment effect.	Simoa™ PD-L1 Reagent Kit	(59)
HNSCC	22	exo-PD-L1 varies in HNSCC by stage, nodal, cell type.	ELISA	(60)
NSCLC	109	exo-PD-L1 level, impact on metastasis & survival.	FCM	(61)
PC	17	PD-L1+ PDAC: shorter post-op survival.	FCM	(62)
Melanoma, NSCLC	18,8	Pre-treat exo-PD-L1 level correlates with immunotherapy response;	ddPCR	(108)
PC	77	exo-PD-L1 is higher in metastatic and links to worse survival.	ELISA	(66)
GC	69	High exoPD-L1, lower survival rate.	ELISA	(8)
GC	80	higher PD-L1 expression have a significantly better prognosis	ELISA	(109)
CC	40	exo-PD-L1 is higher than in NED patients and varies with disease stage.	FCM	(33)
Glioblastoma	21	Circulating exo-PD-L1 DNA correlates with tumor size and tissue PD-L1 levels in blood.	ddPCR	(24)
CRC	192	ExoPD-L1 was almost undetectable	FCM	(70)

NSCLC, non-small cell lung cancer; SCLC, Small Cell Lung Cancer.

HNSCC, Head and neck squamous cell carcinoma; RCC, Renal Cell Carcinoma; HCC, hepatocellular-cancer; CHC, combined hepatocellular cholangiocarcinoma.

EC, endometrial cancer; CC, cervical carcinoma.

PC, Pancreatic Carcinoma; GC, gastric cancer; BC, breast cancer.

CRC, colorectal cancer.

Circulating exo-PD-L1 reflects the immunosuppressive tumor microenvironment and may serve as an indicator of ICB responsiveness (8, 9, 32). Nonetheless, its predictive value varies across cancer types due to tumor heterogeneity and individual immune differences. These challenges are elaborated in subsequent sections.

Research on exo-PD-L1 is most advanced in melanoma, where early increases in circulating exo-PD-L1 during PD-1 blockade have been shown to distinguish responders from non-responders, reflecting T cell reactivation. In metastatic melanoma, a 2.43-fold increase in exo-PD-L1 correlates with favorable outcomes, despite the lack of consistent association with tumor PD-L1 expression (9). Additionally, metastatic melanoma patients exhibit higher exo-PD-L1 levels than healthy donors. Similar patterns have been observed in NSCLC, supporting its cross-tumor relevance as a treatment response biomarker.

Further studies show that melanoma patients with pre-treatment exo-PD-L1 levels below 25.96 pg/mL have better survival rates (56). Post-treatment, exo-PD-L1 levels rise more significantly in responders than in non-responders (32)., whereas total or non-exosomal PD-L1 levels show no such distinction (9, 57). An early increase in exo-PD-L1 during therapy may serve as a dynamic marker for patient stratification (57). Moreover, exo-PD-L1 levels correlate with tumor burden and IFN-γ levels, reinforcing their value in prognosis and treatment monitoring (9, 38).

### 4.1 Exo-PD-L1 in NSCLC

In NSCLC studies, exo-PD-L1 in patient plasma strongly correlates with positive tumor PD-L1 expression (14). Exosomal miR-5684 and miR-125b-5p are significantly lower in patients’ peripheral blood compared to healthy donors (20). Exo-PD-L1 levels were significantly higher in NSCLC patients compared to healthy donors, especially in those with advanced tumor features, while sPD-L1 levels showed no significant difference between the two groups (58). In another NSCLC report, a fold change in exo-PD-L1 of ≥ 1.86 was associated with better therapeutic outcomes and overall survival (OS) (59).

### 4.2 Exo-PD-L1 in cervical cancer

In cervical cancer patients, non-recurrent individuals had higher levels of tumor-enriched CD3- exoPD-L1 before treatment, which significantly decreased five weeks post-treatment (60, 61). Conversely, recurrent patients showed an increase in tumor-enriched exoPD-L1 and a decrease in CD3+ exoPD-L1 levels at week 5 of treatment (61).

### 4.3 Exo-PD-L1 in pancreatic ductal adenocarcinoma

In pancreatic ductal adenocarcinoma, exo-PD-L1 is associated with poor prognosis (62). Higher levels are linked to unresectable tumors and shorter survival. miRNA-196a, miRNA-1246, miRNA-191, miRNA-21, miR-451a, and miRNA-483-3p are elevated in exosomes or liquid biopsies (63–65). In NSCLC, higher extracellular vesicle PD-L1 mRNA levels before treatment are related to better responses. In pancreatic cancer, serum exo-PD-L1 is higher in metastatic patients, and elevated levels mean worse survival (66).

### 4.4 Exo-PD-L1 in gastric cancer

In gastric cancer, preoperative soluble PD-L1 levels aren’t linked to clinical outcomes (8). Higher circulating PD-L1 may indicate malignancy, but its correlation with staging and prognosis is inconsistent. In advanced cases, tumor tissue PD-L1 is higher than in healthy tissues and ties to differentiation and lymph node metastasis. In adenocarcinoma, high PD-L1 expression means better prognosis. Exo-PD-L1 signals mean poor post-treatment outcomes and is an early gastric adenocarcinoma prognostic factor related to tumor staging (66).

### 4.5 Exo-PD-L1 in head and neck squamous cell carcinoma

In head and neck squamous cell carcinoma studies, exo-PD-L1 serves as a marker of poor outcomes following surgery or chemoradiotherapy (58). The level of exo-PD-L1 correlates with disease activity (33). However, studies on glioblastoma indicate that exo-PD-L1 levels cannot distinguish glioblastoma patients from healthy donors (24, 34, 35).

### 4.6 Exo-PD-L1 in “cold tumor”

Although research on exo-PD-L1 has provided valuable insights in many cancer types, its role in cold tumors like ovarian cancer remains poorly understood. Ovarian cancer, often described as a ‘cold tumor’ due to its lack of immune cell infiltration, shows minimal response to immune checkpoint blockade (ICB) therapies. Preliminary studies on exo-PD-L1 in ovarian cancer suggest that low levels of exo-PD-L1 may contribute to this poor response, but further research is needed to better understand its potential as a biomarker in this setting. Ovarian cancer’s limited immune response is thought to be due to the absence of a robust T-cell infiltrate, which limits the effectiveness of PD-1/PD-L1 blockade therapies. Some preliminary data suggest that ovarian cancer patients exhibit relatively low exo-PD-L1 levels, which might contribute to the poor response observed in clinical settings (54, 55). However, further research is needed to understand the role of exo-PD-L1 in this and other less-responsive cancers.

Accurate diagnosis, staging, and prognosis assessment are crucial for enhancing immune therapy response. Immunohistochemistry (IHC) detection of tumor PD-L1 expression, tumor mutational burden (TMB) and microsatellite instability (MSI) are the most commonly used biomarkers for predicting immune therapy response (67–69). However, methodological differences among studies and the dynamic regulation of PD-L1 expression have led to conflicting evidence, limiting the use of tumor PD-L1 expression as an exclusionary biomarker (69). Thus, liquid biopsy may reemerge as a non-invasive tool for screening candidate factors influencing clinical outcomes of immune therapy. Exo-PD-L1 provides a platform to assess cancer patients’ immune status non-invasively. However, correlating exo-PD-L1 levels with tumor PD-L1 expression remains challenging.

In conclusion, exo-PD-L1 shows potential in tumor staging, detection, and aiding ICB therapy. However, a universal standard for evaluation does not exist across different cancers. Future research must focus on analyzing the relationship between PD-L1 and exo-PD-L1 in various cancer tissues, exploring the statistical relationships of exoPD-L1 in different tumors, standardizing detection techniques, and establishing critical ratio for exoPD-L1 and tumor PD-L1 values to differentiate patient conditions and prognosis (70). Additionally, translating these findings into clinical practice requires overcoming significant challenges in standardizing exo-PD-L1 measurement and interpretation across different tumor types and clinical settings.

## 5 Exosomal PD-L1 detection technologies

Peripheral blood is crucial for liquid biopsy, aiding in disease subtyping pre - treatment and minimal residual disease monitoring post - therapy. Exosomes in peripheral blood have higher concentrations and better stability, biocompatibility, low immunogenicity, and minimal toxicity than ctDNA and CTCs. As lipid bilayer vesicles, they’re less affected by the environment and degradation, helping distinguish tumor-derived exosomes from normal ones. In cancer patients, exosomes from serum are analyzed for immune-suppressive molecules like PD-L1, FasL, TRAIL, IL-10, and TGF-β1. In ovarian cancer, ascites and plasma can be analyzed for soluble cytokines via ELISA and CBA, and exosomes from them examined for immune checkpoint molecules like exo-PD-L1 or exo-Gal-9 (12).

### 5.1 Exosome isolation methods

Exosomes are small extracellular vesicles that play a crucial role in intercellular communication, and their detection and analysis are essential for various clinical applications. Due to their small size and high heterogeneity, ultracentrifugation has long been the predominant method for exosome separation. This technique involves differential centrifugation to remove cells and debris, followed by high-speed centrifugation (up to 100,000 g) to isolate microvesicles, ultimately obtaining purified exosomes. However, the time-consuming nature of this method in clinical settings has led to the development of faster techniques, such as affinity-based purification kits. These kits employ antibodies coupled with magnetic beads targeting exosome surface markers (e.g., CD63, CD9, CD81) or proteins with T-cell immunoglobulin and mucin domains (e.g., TIM4) for efficient exosome isolation (71). Recent advancements in flow cytometry, such as Nano-FACS, have been adapted for exosome analysis, validating the potential clinical applications of these rapid separation methods for exosomes and their surface markers, including PD-L1 (71).

### 5.2 PD-L1 detection techniques

Current methods for detecting PD-L1 on tumor-derived exosomes primarily involve ultracentrifugation coupled with ELISA, though these suffer from low efficiency and sensitivity, limiting widespread clinical use (72, 73). Recent advancements include the development of HOLMES-exoPD-L1, a homogeneous, low-volume, high-efficiency, and high-sensitivity quantitative method for exosome PD-L1 detection (74). This technique combines PD-L1 aptamers with HOLMES technology, eliminating the need for separation and offering significantly higher sensitivity and faster detection compared to traditional ELISA methods (74, 75).

In addition, there exist several conventional techniques for the detection of exo-PD-L1 (exo-PD-L1). Electron microscopy and immunoelectron microscopy can serve as qualitative methods to confirm the presence of exoPD-L1. The percentage of exoPD-L1 can be measured using nanoscale flow cytometry or conventional flow cytometry with the aid of magnetic or latex beads. The relative quantification of PD-L1 levels is often assessed through relative fluorescence intensity. Western blotting is used to evaluate the total PD-L1 protein levels within exosomes. Both flow cytometry and immunoblotting provide semi-quantitative measurements. Absolute quantification can be achieved through enzyme-linked immunosorbent assay (ELISA). However, it is important to note that Western blotting and ELISA have detection limits and may not be suitable for detecting low abundance of exoPD-L1 in the early stages of cancer (76). In addition to Western blotting (WB) and ELISA, devices such as the Exo-Counter can directly quantify exosomes expressing specific membrane molecules using as little as 10–50 microliters of plasma or bodily fluid samples without the need for exosome isolation. These advancements hold promise for meeting the future demands of rapid clinical diagnostics (77, 78).

To address ELISA limitations, the nano plasmonic extravesicular (nPLEX) assay has been developed, utilizing modified surface plasmon resonance (SPR) biosensors and compact SPR biosensors (79, 80). This approach enables real-time detection of exoPD-L1 in 50 µL serum samples, demonstrating enhanced detection sensitivity (80).

Additionally, a rapid and precise method for detecting exo-PD-L1 directly from clinical samples has been established using Fe3O4@TiO2 separation and surface-enhanced Raman scattering (SERS) immunoassays. This approach significantly enhances both the separation efficiency and detection sensitivity of exosomes (54, 81).

**Figure 2:**
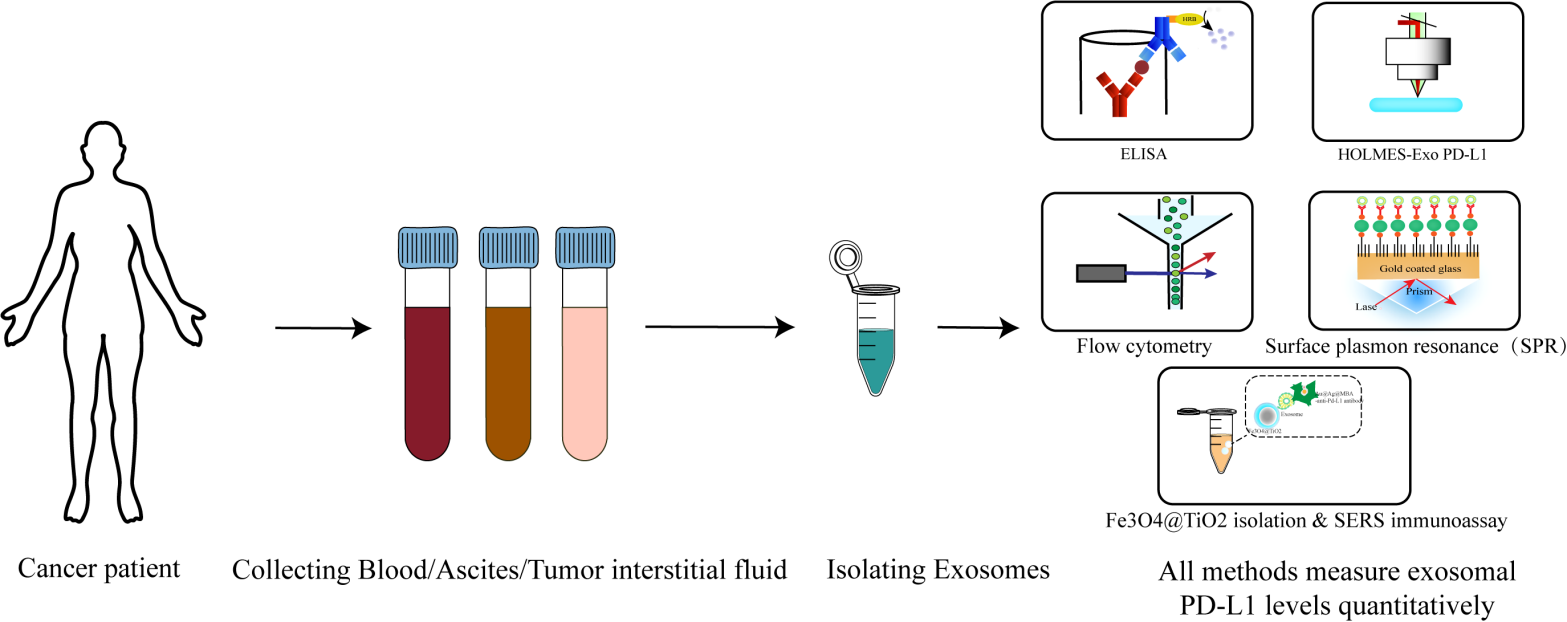
Detection process of exo-PD-L1: from sample collection to modern detection techniques. The diagram outlines the process for detecting exo-PD-L1, a biomarker for cancer diagnostics. The workflow begins with the collection of patient samples, which may include peripheral blood, tumor tissue fluid, or ascites (in cases such as ovarian cancer) (1). Following sample collection, exosomes are isolated using various methods such as ultracentrifugation, affinity-based purification, or advanced techniques like Nano-FACS (2). Once exosomes are purified, the presence of PD-L1 on their surface is detected using several techniques. Traditional methods include enzyme-linked immunosorbent assay (ELISA). Recent advancements feature high-sensitivity approaches such as HOLMES-exo-PD-L1, nano plasmonic extravesicular (nPLEX) assay, and surface-enhanced Raman scattering (SERS) immunoassays (4). These modern techniques offer improved detection sensitivity and efficiency. The figure highlights the steps from sample collection through exosome isolation to the detection of exo-PD-L1, reflecting the current state-of-the-art in exosome-based cancer diagnostics.

Despite challenges posed by inconsistent exosome separation procedures, quality control, and storage methods, emerging microfluidic-based separation technologies (e.g., nanoparticle platforms) are being investigated as next-generation solutions for effective exosome isolation (82–85). As these technical challenges are addressed, the potential of exosomes in cancer therapy is expected to be further realized (85) (**Figure 2**; **Table 2**).

**Table 2:** Comparison of different detection methods for exo-PD-L1.

Method	Strengths	Limitations
Enzyme-Linked Immunosorbent Assay (ELISA)	✔ Widely used and standardized ✔ suitable for various sample types ✔ high specificity ✔ quantitative analysis.	✔ low sensitivity ✔ lengthy reaction time ✔ requires larger sample volumes
HOLMES-exo-PD-L1 Method	✔ high sensitivity and recognition efficiency ✔ rapid, non-invasive detection; ✔ requires minimal sample volume ✔ easy to operate.	✔ new technology with limited clinical application experience ✔ rely on the availability of aptamers
Flow Cytometry (FCM)	✔ High-throughput ✔ multiparameter detection of multiple markers	✔ Requires equipment and is costly; ✔ need labeling ✔ not suited for real-time monitoring ✔ Instrument acquisition and maintenance entail substantial financial investment
Surface Plasmon Resonance (SPR)	✔ Real-time ✔ label-free detection ✔ high sensitivity ✔ suited for analyzing biomolecular interactions	✔ High-cost equipment and complex operation ✔ Not suited for large-scale applications due to size and expense constraints ✔ High operational costs restrict applicability in routine diagnostics
Fe3O4@TiO2 isolation and SERS immunoassay	✔ Extremely high sensitivity ✔ allowing for single exosome-level detection ✔ rapid detection with minimal sample volume ✔ non-destructive monitoring	✔ Requires specialized nanoparticles and high-cost spectroscopic equipment ✔ data analysis is complex, technically demanding ✔ High cost and complex data interpretation hinder clinical implementation

## 6 Therapeutic applications and targeting strategies

By alleviating the suppression of T cells, reducing exosome secretion can potentially restore anti-tumor T cell responses. However, therapies specifically targeting exosomal PD-L1 (exo-PD-L1) are still in their nascent stages, with no direct exo-PD-L1-targeting treatments currently available. Current approaches predominantly focus on inhibiting exosome production, thereby indirectly lowering exo-PD-L1 levels.

### 6.1 Inhibition of exosome biogenesis and secretion

Exosome release can be inhibited using antibodies, chemical inhibitors, and genetic manipulation, enhancing cancer treatment efficacy (86, 87). Exosome formation, cargo sorting, and secretion rely on the Endosomal Sorting Complex Required for Transport (ESCRT) mechanism. High-throughput screening of 4,580 compounds identified five effective inhibitors: tipifarnib, naftifine, clomipramine, ketoconazole, and miconazole (88). These suppress exosome production by downregulating ESCRT-dependent proteins (88–90).Dimethyl amiloride (DMA) inhibits exosome release by targeting H+/Na+ and Na+/Ca2+ channels, reducing exosome-induced immunosuppression and enhancing anti-tumor efficacy, making it a promising chemotherapy approach (91). GW4869 and spiroepoxide inhibit neutral sphingomyelinase (nSMase) via ESCRT-independent mechanisms (92), blocking exosome secretion. As ceramide biosynthesis inhibitors, GW4869 suppresses exosome secretion in 293T cells and inhibits ovarian cancer exosome release and cell invasion (93).

### 6.2 Combination with immune checkpoint inhibitors

Combining exosome inhibition with immune checkpoint blockade has emerged as a promising therapeutic strategy to enhance antitumor immunity. By reducing circulating exo-PD-L1 levels, such combinations may enhance the accessibility and efficacy of anti-PD-1/PD-L1 antibodies within the tumor microenvironment. In breast cancer mouse models, treatment with sulfamethoxazole, macitentan, and anti–PD-L1 antibodies significantly decreased plasma exo-PD-L1 levels, reactivated cytotoxic T cells, and led to reduced tumor growth and metastasis (94–96). These findings suggest that exosome-mediated immune suppression may be reversible and that targeting exo-PD-L1 could enhance responsiveness to ICIs.

### 6.3 Alternative physical approaches

In addition to pharmacologic inhibition, several physical approaches have been investigated for exosome elimination. Autophagy induction has been shown to suppress exosome biogenesis by promoting degradation of multivesicular bodies. On the other hand, extracorporeal removal techniques, such as blood purification systems and ultrafiltration, offer non-invasive methods to physically eliminate exosomes from circulation. These technologies are still in preclinical evaluation but have demonstrated potential advantages over drug-based therapies, including lower systemic toxicity and broader applicability across tumor types (97). Nevertheless, their specificity for exo-PD-L1 remains to be established (97).

Regulating exo-PD-L1 expression is complex. Pre-clinical studies on exo-PD-L1-targeting strategies are promising, but more research is required to solve challenges and explore their combined clinical effectiveness (4). Further investigations are necessary to identify precise, clinically feasible approaches that can modulate exo-PD-L1 without disrupting essential exosome functions.

As these strategies advance toward clinical translation, both efficacy and the spectrum of on- and off-target effects must be rigorously benchmarked. First-generation chemical blockers of exosome biogenesis—such as the nSMase2 inhibitor GW4869 and the RAB27A-JFC1 interaction disruptor Nexinhib-20—display only cell-line-restricted activity and fail to curb vesicle release in prostate (PC3) and other cancer models or to produce an *in-vivo* benefit in the MC38 syngeneic tumor (90, 98). Compounds that more globally perturb vesicle trafficking, for example dimethyl-amiloride, also interfere with lipid metabolism or ion transport and may introduce unintended immunotoxicity (99). Notably, small-molecule nSMase2 blockade with GW4869 has been linked to phosphatidylserine-dependent cytotoxicity in myeloma cells and memory impairment in mice, while Nexinhib-20 suppresses β_2_-integrin activation in neutrophils, raising infection or bleeding concerns. Dimethyl-amiloride (100), a classic Na^+^/H^+^-exchanger blocker, perturbs cardiomyocyte ion homeostasis at micromolar doses (101–103). These emerging data underscore the need for structure-guided optimization and rigorous toxicology before first-in-human trials. Likewise, bulk physical-removal approaches—such as extracorporeal plasma exchange—risk indiscriminately depleting exosomes with homeostatic roles in immune regulation or tissue repair (104). Therefore, future work should aim to improve the specificity, tolerability, and context-dependent application of these interventions to ensure clinical safety and efficacy.

## 7 Future perspectives, challenges, and conclusions

Exosomal PD-L1 (exo-PD-L1) plays a critical role in tumor initiation, progression, and immune suppression. Tumor-derived exo-PD-L1 binds to PD-1 receptors on T cells, thereby inhibiting anti-tumor immune responses both locally and systemically. Moreover, exo-PD-L1 may serve as a molecular sink for immune checkpoint inhibitors by sequestering anti-PD-L1 antibodies in the circulation, limiting their effective concentration within the tumor microenvironment. By integrating exosome analysis into personalized treatment strategies, clinicians can tailor ICI treatment plans based on exo-PD-L1 levels, improving treatment efficiency and accurately predicting patient responses.

### 7.1 Limitations and challenges

Despite its emerging clinical value, the use of exosomal PD-L1 (exo-PD-L1) as a predictive biomarker for immune checkpoint blockade (ICB) therapy remains constrained by several critical limitations. One major challenge lies in its cellular origin: exo-PD-L1 is not exclusively secreted by tumor cells but may also be derived from immune and stromal cells, thereby compromising its tumor specificity and interpretative precision (75). Furthermore, the relationship between exo-PD-L1 levels and therapeutic response has proven inconsistent across different cancer types, undermining its reliability as a universal biomarker (76). Technical and practical hurdles also present significant barriers to clinical translation. The absence of standardized isolation and quantification protocols contributes to inter-study variability, while the lack of established clinical thresholds impedes its integration into routine diagnostic workflows (56, 77, 78). In addition, many of the most sensitive and specific detection methods—such as surface plasmon resonance (SPR) biosensing, SERS-based assays, and nanoparticle-enhanced platforms—require sophisticated instrumentation, specialized reagents, and high operational costs, which substantially limit their accessibility in standard clinical settings (**Table 2**). Together, these issues highlight the need for methodological harmonization, cost reduction, and further clinical validation before exo-PD-L1 can be reliably adopted in precision oncology.

### 7.2 Exo-PD-L1/IHC-PD-L1 ratio as a predictive model for immunotherapy

**Figure 3:**
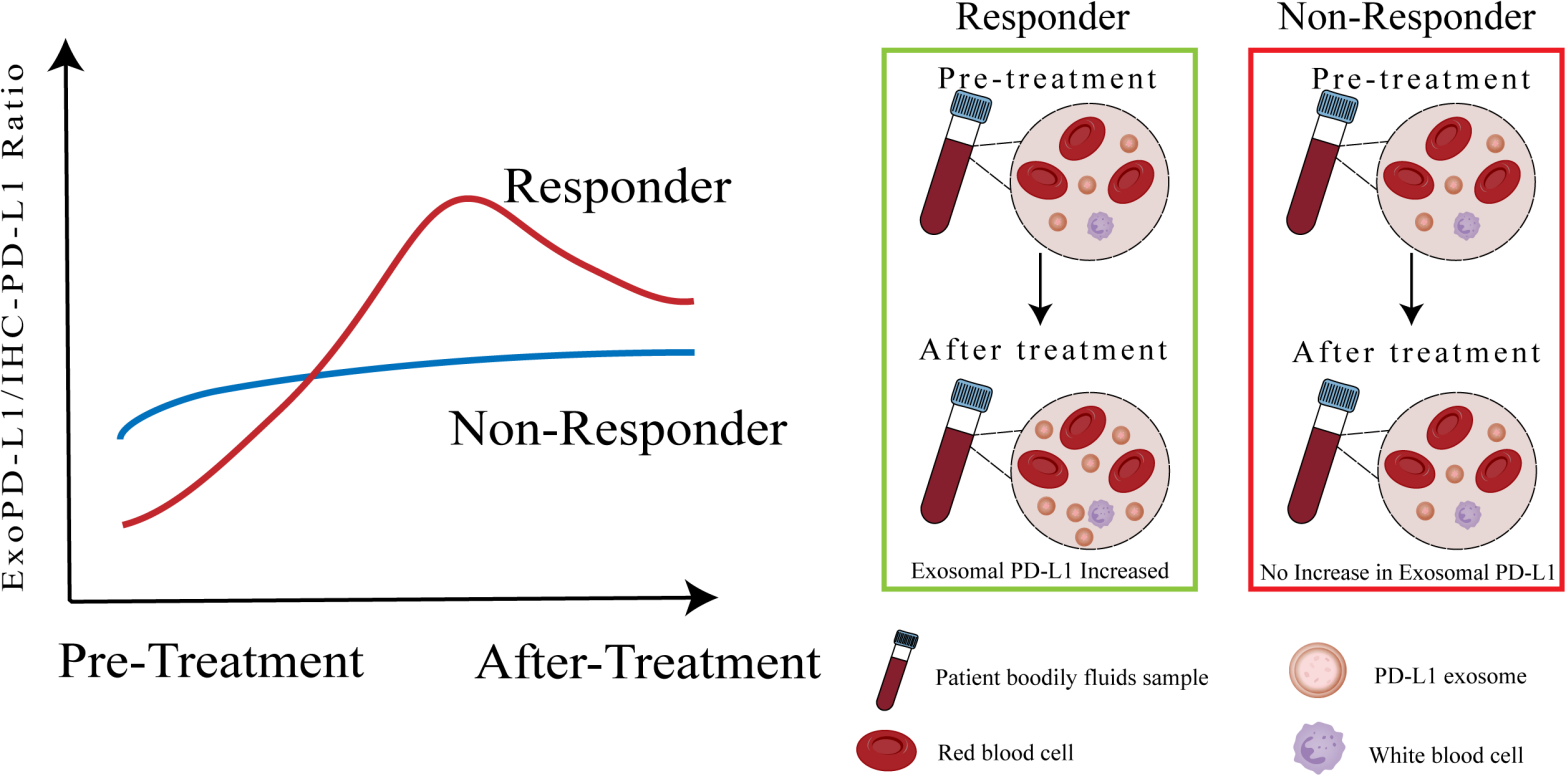
Exo-PD-L1/IHC-PD-L1 ratio as a potential biomarker for predicting immune checkpoint blockade therapy response. This figure illustrates the proposed utility of the ratio between circulating exosomal PD-L1 and tumor PD-L1 expression (exo-PD-L1/IHC-PD-L1) as a dynamic and potentially more informative biomarker for predicting clinical response to ICB therapy. Unlike static, single-site measurements, this ratio may better capture the interplay between localized immune escape and systemic immunomodulation. In responders, a low baseline ratio followed by a marked post-treatment increase in exosomal PD-L1 reflects enhanced immune activation and treatment efficacy. In contrast, non-responders tend to display a high baseline ratio with minimal change after therapy, indicating persistent immune suppression. This ratio-based model may enable early patient stratification and guide the design of personalized immunotherapeutic strategies, though further validation is needed.

An important avenue for future research is to explore the prognostic and predictive potential of the exosomal PD-L1 to tumor PD-L1 ratio (exo-PD-L1/IHC-PD-L1) in the context of immune checkpoint inhibitor (ICI) therapy. While exosomal PD-L1 and tumor PD-L1 have each been individually investigated as biomarkers, this ratio may offer enhanced discriminatory power by integrating both systemic and tumor-localized immunosuppressive cues. Incorporating this dynamic ratio into future stratification models could improve patient selection and inform more personalized immunotherapeutic strategies. (**Figure 3**) Additionally, a comprehensive evaluation of tissue PD-L1, exo-PD-L1, and soluble PD-L1 is essential for companion diagnostics. Emerging studies suggest that tumor metabolic regulation and nanotechnology-based strategies could influence PD-L1 expression and secretion, offering potential tools to indirectly modulate exo-PD-L1 levels (36, 105–107).

### 7.3 Applications of exo-PD-L1 in precision oncology

A fascinating avenue is the potential of exo-PD-L1 as an early warning system for predicting organ-specific metastasis. By analyzing exo-PD-L1 levels from body fluids, researchers could potentially infer which organ may be the next site of metastatic spread. However, this approach still requires extensive validation, as identifying definitive biomarkers for organ-specific metastasis remains challenging. Additionally, monitoring exo-PD-L1 dynamics could support combination therapies that reprogram cold tumors into responsive ones, improving ICB therapy outcomes in resistant cancers (36).

### 7.4 Conclusion

In advancing anti-PD-1/PD-L1 immune checkpoint blockade therapies, it is essential to consider both PD-L1 on tumor cell surfaces and exo-PD-L1 levels for a comprehensive evaluation of patient conditions. In conclusion, exo-PD-L1 shows great promise as a multifaceted biomarker in immune checkpoint blockade therapy. By integrating exo-PD-L1 levels into clinical decision-making, we can potentially refine personalized treatment regimens, enhance the precision of immunotherapies, and ultimately improve patient outcomes.
